# Single-bone versus both-bone plating of unstable paediatric both-bone forearm fractures. A randomized controlled clinical trial

**DOI:** 10.1007/s00264-021-05097-z

**Published:** 2021-06-12

**Authors:** Mohamed Khaled, Amr A. Fadle, Ahmed Khalil Attia, Andrew Sami, Abdelkhalek Hafez, Nariman Abol Oyoun

**Affiliations:** 1 1Orthopedic Department, Assiut University, Assiut, Egypt; 2 Orthopedic Surgery Department, Hamad Medical Corporation, PO Box 3050, Doha, Qatar

**Keywords:** Single-bone fixation, Both-bone fixation, Ulna ORIF, Pediatric forearm fractures

## Abstract

**Purpose:**

This clinical trial compares the functional and radiological outcomes of single-bone fixation to both-bone fixation of unstable paediatric both-bone forearm fractures.

**Methods:**

This individually randomized two-group parallel clinical trial was performed following the Consolidated Standards of Reporting Trials (CONSORT) statement at a single academic tertiary medical centre with an established paediatric orthopaedics unit. All children aged between nine and 15 years who presented to the emergency department at Assiut university with unstable diaphyseal, both-bone forearm fractures requiring surgical intervention between November 1, 2018, and February 28, 2020, were screened for eligibility against the inclusion and exclusion criteria. Inclusion criteria were diaphyseal unstable fractures defined as shaft fractures between the distal and proximal metaphyses with an angulation of > 10°, and/or malrotation of > 30°, and/or displacement > 10 mm after attempted closed reduction. Exclusion criteria included open fractures, Galeazzi fractures, Monteggia fractures, radial head fractures, and associated neurovascular injuries. Patients who met the inclusion criteria were randomized to either the single-bone fixation group (intervention) or the both-bone fixation group (control). Primary outcomes were forearm range of motion and fracture union, while secondary outcomes were forearm function (price criteria), radius re-angulation, wrist and elbow range of motion, and surgical time

**Results:**

A total of 50 children were included. Out of these 50 children, 25 were randomized to either arm of the study. All children in either group received the treatment assigned by randomization. Fifty (100%) children were available for final follow-up at six months post-operatively. The mean age of single-bone and both-bone fixation groups was 11.48 ± 1.93 and 13 ± 1.75 years, respectively, with a statistically significant difference (*p* = 0.006). There were no statistically significant differences in gender, laterality, affection of the dominant hand, or mode of trauma between single-bone and both-bone fixation groups. All patients in both groups achieved fracture union. There mean radius re-angulation of the single-bone fixation groups was 5.36 ± 4.39 (0–20) degrees, while there was no radius re-angulation in the both-bone fixation group, with a statistically significant difference (*p* < 0.001). The time to union in the single-bone group was 6.28 ± 1.51 weeks, while the time to union in the both-bone fixation group was 6.64 ± 1.75 weeks, with no statistically significant difference (*p* = 0.44). There were no infections or refractures in either group. In the single-bone fixation group, 24 (96%) patients have regained their full forearm ROM (loss of ROM < 15°), while only one (4%) patient lost between 15 and 30° of ROM. In the both-bone fixation group, 23 (92%) patients have regained their full forearm ROM (loss of ROM < 15°), while only two (8%) patients lost between 15 and 30° of ROM. There was no statistically significant difference between groups in loss of forearm ROM (*p* = 0.55). All patients in both groups regained full ROM of their elbow and wrist joints. On price grading, 24 (96%) and 23 (92%) patients who underwent single bone fixation and both-bone fixation scored excellent, respectively. Only one (4%) patient in the single-bone fixation group and two (8%) patients in the both-bone fixation group scored good, with no statistically significant difference in price score between groups (*p* = 0.49). The majority of the patients from both groups had no pain on the numerical pain scale; 22 (88%) patients in the single-bone fixation group and 21 (84%) patients in the both-bone fixation groups, with no statistically significant difference between groups (*p* = 0.38). The single-bone fixation group had a significantly shorter mean operative time in comparison to both-bones plating (43.60 ± 6.21 vs. 88.60 ± 10.56 (min); *p* < 0.001).

**Conclusion:**

Single-bone ulna open reduction and plate fixation and casting are safe and had a significantly shorter operative time than both-bone fixation. However, single-bone ORIF had a higher risk radius re-angulation, alas clinically acceptable. Both groups had equally excellent functional outcomes, forearm ROM, and union rates with no complications or refractures. Long-term studies are required.

## Introduction

Radius and ulna fractures, or both-bone forearm fractures, are the third most common injuries in children [1], and diaphyseal forearm fractures are common injuries that represent between 3 and 6 percent of all paediatric fractures [2]. An important anatomical feature of the forearm is the interosseous membrane, which is a fibrous structure with an oblique orientation from the radius to the ulna [3]. It upholds the interosseous space between the radius and ulna during forearm pronation and supination and actively transfers forces between forearm bones and acts as a stiff structure with elastic properties that is able to sustain large loads [3]. Due to the forearm’s unique features as a joint, fractures of the radius and ulna should be approached like other articular fractures [4].

While closed reduction and cast immobilization remain the gold standard treatment for minimally displaced and stable pediatric forearm fractures in younger children, children of nine  years of age or older may tolerate no more than 8–10° of angular deformation in middle-third fractures, at most 30° in rotational deformation and no more than 100% of displacement [5]. Twenty degrees of fracture angulation in the middle 1/3 of the forearm was reported to cause obvious limitation in forearm pronation-supination in a cadaveric study [6]. The more stringent criteria for an acceptable alignment in older children also stems from reduced remodeling potential. Children younger than nine years old have a remodeling potential of up to 20° by skeletal maturity. Fractures closer to the distal physis show the greatest remodeling. On the other hand, children older than nine years old with forearm shaft fractures do not predictably remodel to a similar degree [7, 8].

The necessity of fixing both radius and ulna has been questioned, and a few studies have explored the option of single-bone fixation of either the radius or the ulna. They reported that single-bone intramedullary fixation was safe and led to good functional outcomes. However, except for one randomized controlled trial (RCT) [9], most of these studies were either retrospective [10–18] or lacked a control group [19]. To the best of our knowledge, our study is the first RCT to compare open reduction and internal fixation (ORIF) of the ulna alone to ORIF of both bones.

### Purpose

The current study aims to compare the outcomes of single-bone fixation to both-bone fixation in unstable diaphyseal both-bone forearm fractures in children. The null hypothesis was that fixation of only one bone has similar results to fixation of both bones.

## Methods

### Trail design and participants

This individually randomized two-group parallel clinical trial was performed following the Consolidated Standards of Reporting Trials (CONSORT) statement [20] at a single academic tertiary medical centre with an established pediatric orthopedics unit. The trial was approved by the Ethics Committee at Assiut University and conducted according to the Helsinki declaration.

All children aged between nine and 15 years who presented to the emergency department at XX university with unstable diaphyseal, both-bone forearm fractures requiring surgical intervention between November 1, 2018, and February 28, 2020, were screened for eligibility against the inclusion and exclusion criteria (Table 1).

**Table 1:** Inclusion and exclusion criteria

Inclusion criteria	Exclusion criteria
• Age between 9 and 15 years	• Ipsilateral upper limb fractures and/or dislocation
• Unilateral or bilateral unstable mid-shaft both-bone forearm fracture	• Open fractures • Stable fractures
• Closed fractures within seven days from injury	• Pathological fractures
• Patients whose parents or legal guardians are willing to provide their consent to participate	• Comminuted forearm shaft fractures • Polytrauma patients
	• Associated nerve or vascular injury requiring repair
	• Monteggia and Galeazzi fracture dislocations
	• Metabolic bone disease
	• Previous ipsilateral upper limb surgery
	• Metaphysis-diaphysis junction fractures
	• Associated radial head fracture
	• Patients whose parents or legal guardians declined to participate

Diaphyseal fractures were defined as shaft fractures between the distal and proximal metaphyses. Unstable fractures were defined as fractures with an angulation of > 10°, and/or malrotation of > 30°, and/or displacement > 10 mm after attempted closed reduction [9, 18]. Written informed consent was obtained for participation from all parents/legal guardians and ascent from all children aged 12 years and older. Patients who met the inclusion criteria were randomized to either the single-bone (ulna) fixation group (intervention) or the both-bone (radius and ulna) fixation group (control). Table 2 highlights the research question.

**Table 2:** Research question (PICO)

Population	9–15 years old children with unstable diaphyseal both-bone forearm fractures
Intervention	Single-bone ORIF
Control	Both-bone ORIF
Outcomes	Primary outcomes
	(1) Forearm range of motion
	(2) Fracture union
	Secondary outcomes
	(1) forearm function (price criteria)
	(2) radius re-angulation
	(3) Wrist and elbow range of motion
	(4) Surgical time

### Outcome measures

The data collected were demographics (age, gender, laterality, dominance, and mode of trauma), management characteristics, and outcome measures. Patients in both groups were evaluated and followed by the same senior paediatric orthopaedic surgeon (MK) during the study period.

Fracture union was defined as the absence of tenderness on palpation and subjective complaint of pain, painless range of motion (ROM) of forearm rotation, and appearance of bridging callus on follow-up x-ray. ROM of the forearm, wrist, and elbow was measured using a universal, transparent goniometer. In the examination of pronation-supination, the subjects were sitting, adducting arms with the elbows in 90° of flexion. The forearms were in a neutral position, thumbs upwards, and progressively active and passive rotation movement followed with Goniometer measurement [21].Wrist and elbow ROM were compared with those on the contralateral side. Grip strength was measured by a clinical squeezing test and compared to the contralateral limb. Radiographic parameters were angulation, rotation, and displacement. Clinical and radiological measures were correlated with price criteria for both groups of patients in this study [22]. Table 3 highlights price grading criteria.

**Table 3:** Price grading criteria

Grade	Patient complaints	Loss of forearm rotation
Excellent	No complaints with strenuous physical activity	$\le$ 10°
Good	Mild complaints with strenuous physical activity	10 to 30°
Fair	Mild subjective complaints during daily activities	$>$ 30 to 90°
Poor	Worse than mild subjective complaints during daily activities	$\ge$ 90°

Adapted from Price et al

### Surgical technique

All of the cases in both groups were operated by the same senior paediatric orthopaedic surgeon (MK). All procedures were done in the supine position with the operative limb on the arm side extension under general anesthesia. Pre-operative intravenous Cefazolin was given before inflation of the tourniquet.

#### Single-bone fixation group

After standard prepping and draping, a standard 6-8 cm long, dorsal subcutaneous approach centered over the ulnar fracture was carried out. Open reduction and fixation of the ulna fracture with a 3.5 mm DCP with a minimum of three screws (Synthes, West Chester PA) on either side of the fracture was made. Closed manipulation of the radius was done to restore alignment. Radial alignment and ulna fixation were checked by intra-operative x-ray projections. Up to 10° of radius angulation was considered acceptable. After wound closure, the limb was kept in a well-padded long arm posterior slab covering 2/3 of the forearm circumference with the elbow in 90° flexion and the wrist in a neutral position. The slab was kept for thirty days along with a broad arm sling.

#### Both-bone fixation group

After standard prepping and draping, the radius was tackled first with the standard dorsolateral approach. Open reduction and fixation of the radius fracture with a 3.5 mm DCP with a minimum of three screws (Synthes, West Chester PA) on either side of the fracture was made. The ulna was reduced and fixed through a dorsal approach similar to the single bone fixation group. After wound closure and dressing application, the limb was kept in a broad arm sling with no backslab.

### Post-operative management protocol

The post-operative protocol was standardized for both groups. For the purpose of this study, both groups were assessed preoperatively and on post-operative day one. After discharge, they were assessed at two weeks, four weeks, six weeks, three months, and six months post-operatively. Anteroposterior and lateral plain radiographs were obtained pre-operatively, intra-operatively, and on post-operative day one. After discharge, radiographs were obtained on the two week, four week, six week, three month, and six month outpatient appointments for the single-bone fixation group. In the both-bone fixation group, the two week and four week radiographs were not routinely done as the authors believed they were unnecessary and to minimize radiation exposure. When the fracture was deemed to be united by bridging callus and absence of fracture site tenderness at six weeks, gentle range of motion was encouraged, but contact sports and athletic activities were restricted for an additional three months. The final follow-up assessment was done at 24 weeks.

### Randomization and blinding

A resident physician (AS) not involved in the surgical intervention randomized the study participants according to a computer-generated sequence using Research Randomizer (Version 4.0) [computer software]. The operating surgeon was informed of the allocation at the induction of anaesthesia. The same senior paediatric orthopaedic surgeon examined all the operated cases in both groups at all follow-up appointments and obtained the radiographic and functional outcomes to avoid interobserver bias. Realistically, the surgeons, as well as the participants, could not be blinded to the intervention as the number of scars and radiographs would have revealed the allocation.

### Statistical analysis

The power calculation was done according to forearm supination/pronation ROM limitations. To assess the sample size required, an equivalence test was used to demonstrate the similarity of forearm supination/pronation ROM limitations in both groups. Equivalence between the two groups was defined as a maximum of 15° of loss of motion in the single-bone group [9]. This number is similar to what was reported in a similar clinical trial on elastic nails by Colaris et al. [9]. According to the power calculation, to generate a power of 80%, an alpha of 0.05, and a standard deviation of 15°, each group should consist of 25 patients.

Intention-to-treat (INT) analysis was carried out for all the outcomes. A separate as-treated analysis was not done as all patients received the intervention to which they were randomized.

Data were analyzed using SPSS 25 (IBM, Armonk, New York). Continuous data were expressed as mean ± standard deviation (SD), while nominal data were expressed as frequency (percentage). Chi-squared (*χ*^2^) test was used to compare the nominal data of different groups in the study, while student *t* test was used to compare the mean of different groups. *p* values were considered significant if < 0.05.

## Results

**Fig. 1:**
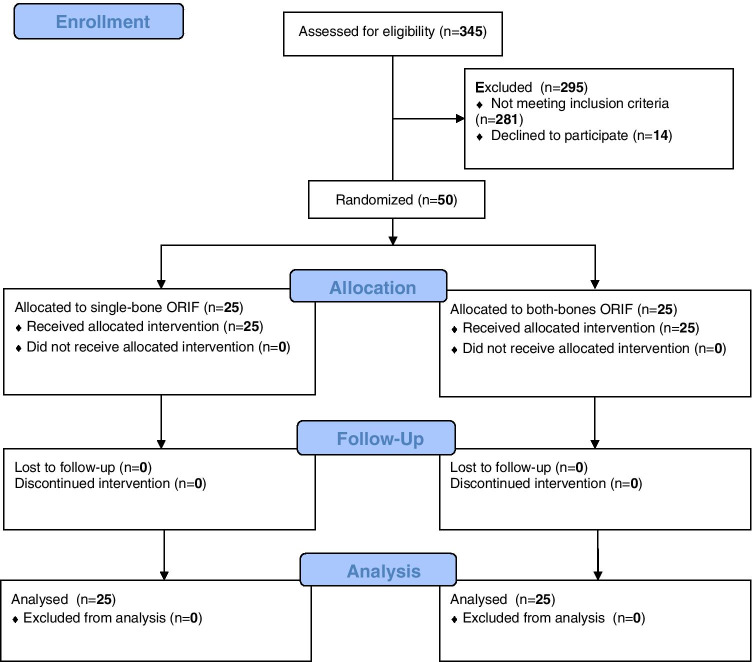
Consolidated Standards of Reporting Trials (CONSORT) flow diagram

Between November 1, 2018, and February 28, 2020, a total of 50 children were included. Out of these 50 children, 25 were randomized to either arm of the study. All children in either group received the treatment assigned by randomization. Fifty (100%) children were available for final follow-up at 6 months postoperatively (Fig. 1).

The mean age of single-bone and both-bone fixation groups was 11.48 ± 1.93 (range: 9–14) and 13 ± 1.75 (range: 9–15) years, respectively, with a statistically significant difference (*p* = 0.006). There were no statistically significant differences in gender, laterality, affection of the dominant hand, or mode of trauma between groups (Table 4).

**Table 4:** Baseline characteristics of single-bone and both-bone fixation groups

	Single-bone ORIF* (*n* = 25)	Both-bone ORIF* (*n* = 25)	*p* value
Age (years)	11.48 ± 1.93	13 ± 1.75	**0.006**
Gender			
Male	24 (96%)	23 (92%)	0.22
Female	1 (4%)	2 (8%)	
Laterality			0.38
Right	12 (48%)	14 (56%)	
Left	13 (52%)	11(44%)	
Injured side			
Dominant	11 (44%)	9 (36%)	0.56
Non-dominant	14 (56%)	16 (64%)	
Mode of trauma			
FOOSH	22 (88%)	20 (80%)	0.37
Heavy object	2 (8%)	3 (12%)	
MVC	1 (4%)	2 (8%)	
Time of surgery			0.11
Same day	19 (76%)	14 (56%)	
Next day	6 (24%)	11 (44%)	

*n* number, *FOOSH* fall on outstretched hand, *MCA* motor vehicle collision. *Data expressed as mean (SD) or frequency (%). *p* value was considered significant if < 0.05

The single-bone fixation group had a significantly shorter mean operative time in comparison to both-bones plating (43.60 ± 6.21 vs. 88.60 ± 10.56 (min); *p* < 0.001). All patients in both groups achieved fracture union. There was no statistically significant difference in time to union between the single-bone and both-bone fixation groups (6.28 ± 1.51 vs. 6.64 ± 1.75 weeks, *p* = 0.44), respectively. There were no infections or refractures in either group (Table 5).

**Table 5:** Operative and outcome data of single-bone and both-bones groups

	Single-bone* (*n* = 25)	Both-bones* (*n* = 25)	*p* value
Operative time (mins)	43.60 ± 6.21	88.60 ± 10.56	** < 0.001**
Union rate	25 (100%)	25 (100%)	–
Time to union (wks)	6.28 ± 1.51	6.64 ± 1.75	0.44
Complications			
Infection	0 (0%)	0 (0%)	–
Re-fracture	0 (0%)	0 (0%)	–

*n* number, *mins* minutes, *wks* weeks. *Data expressed as mean (SD) or frequency (%). – Could not be calculated. *p* value was considered significant if < 0.05

In the single-bone fixation group, 24 (96%) patients have regained their full forearm ROM (loss of ROM < 15°), while 23 (92%) patients have regained their full forearm ROM (loss of ROM < 15°) in the both-bone fixation group, with no statistically significant difference (*p* = 0.55). There mean radius re-angulation of the single-bone fixation groups was 5.36 ± 4.39 (0–20) degrees, while there was no radius re-angulation in the both-bone fixation group, with a statistically significant difference (*p* < 0.001) (Table 6).

**Table 6:** Radiographic and functional outcomes of single-bone vs. both-bone fixation groups

	Single-bone* (*n* = 25)	Both-bones* (*n* = 25)	*p-*value
Loss of forearm (pronation/supination) ROM			0.55
0–15°	24 (96%)	23 (92%)	
16–30°	1 (4%)	2 (8%)	
Radial re-angulation (°)	5.36 ± 4.39	0 ± 0.0	** < 0.001**
Full elbow ROM	25 (100%)	25 (100%)	-
Full wrist ROM	25 (100%)	25 (100%)	-
Price grading [16]			0.49
Excellent	24 (96%)	23 (92%)	
Good	1 (4%)	2 (8%)	
Fair	0 (0%)	0 (0%)	
Poor	0 (0%)	0 (0%)	
Numerical pain scale (NPS)			0.38
No pain	22 (88%)	21 (84%)	
Mild	0 (0%)	2 (8%)	
Moderate	3 (12%)	2 (8%)	
Severe	0 (0%)	0 (0%)	

*n* number, *ROM* range of motion. *Data expressed as mean (SD) or frequency (%). *p* value was considered significant if < 0.05

On price grading, 24 (96%) and 23 (92%) patients who underwent single bone fixation and both-bone fixation scored excellent, respectively. Twenty-two (88%) patients in the single-bone fixation group and 21 (84%) patients in the both-bone fixation groups had no pain, with no statistically significant difference between groups (*p* = 0.38) (Table 6).

## Discussion

**Fig. 2:**
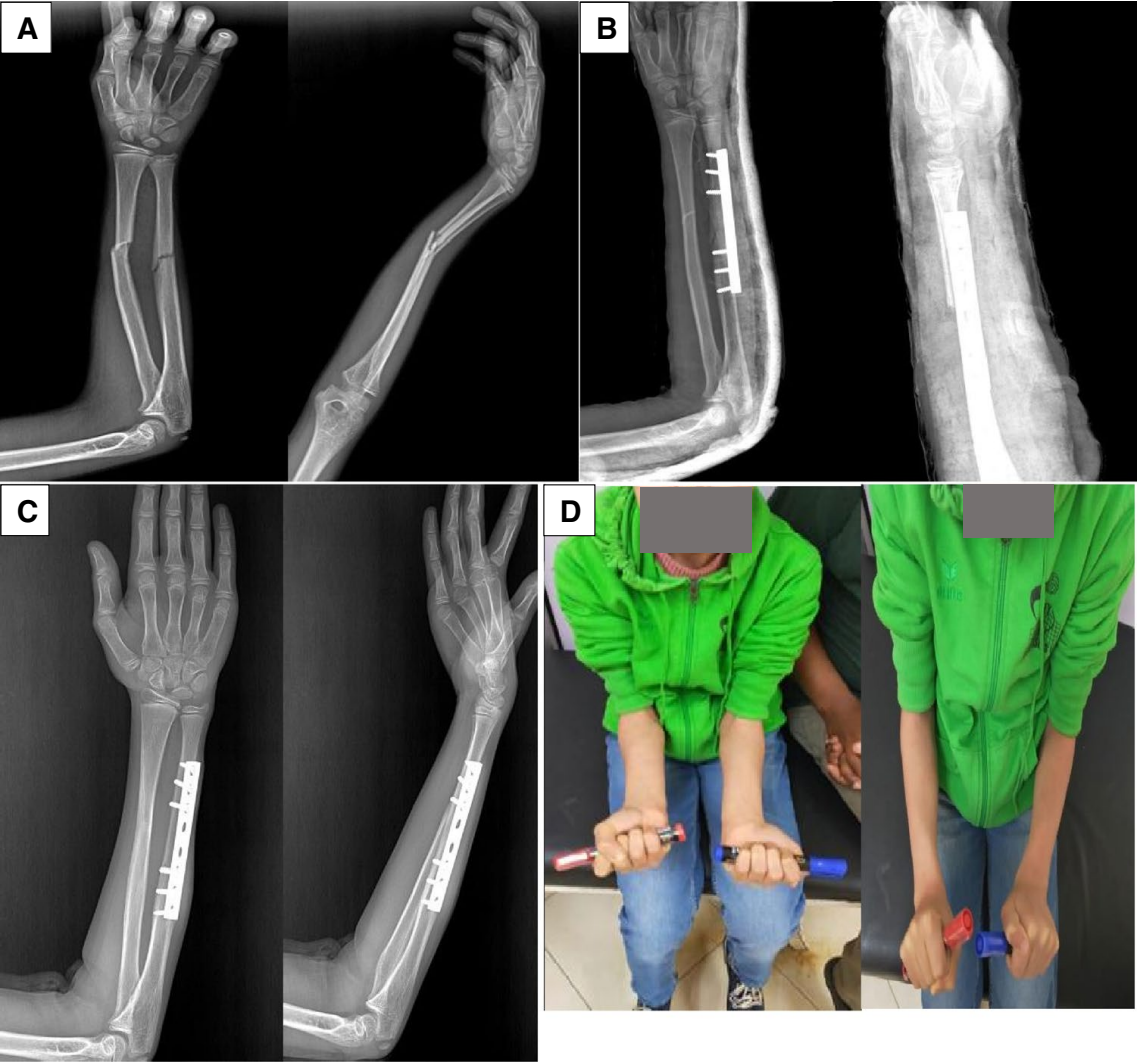
Ten-year-old male child who sustained right both-bone unstable forearm injury following fall down accident. **A**—Pre-operative radiographs showing mid-shaft forearm fracture with Apex volar angulation. **B**—Post –operative x-rays (AP & lateral) showing single-bone ulna fixation with well reduced radius. **C**—X-rays (AP & lateral) at 6-week post-op which confirm radiological union. **D**—Clinical photograph showing comparable forearm range of motion

**Fig. 3:**
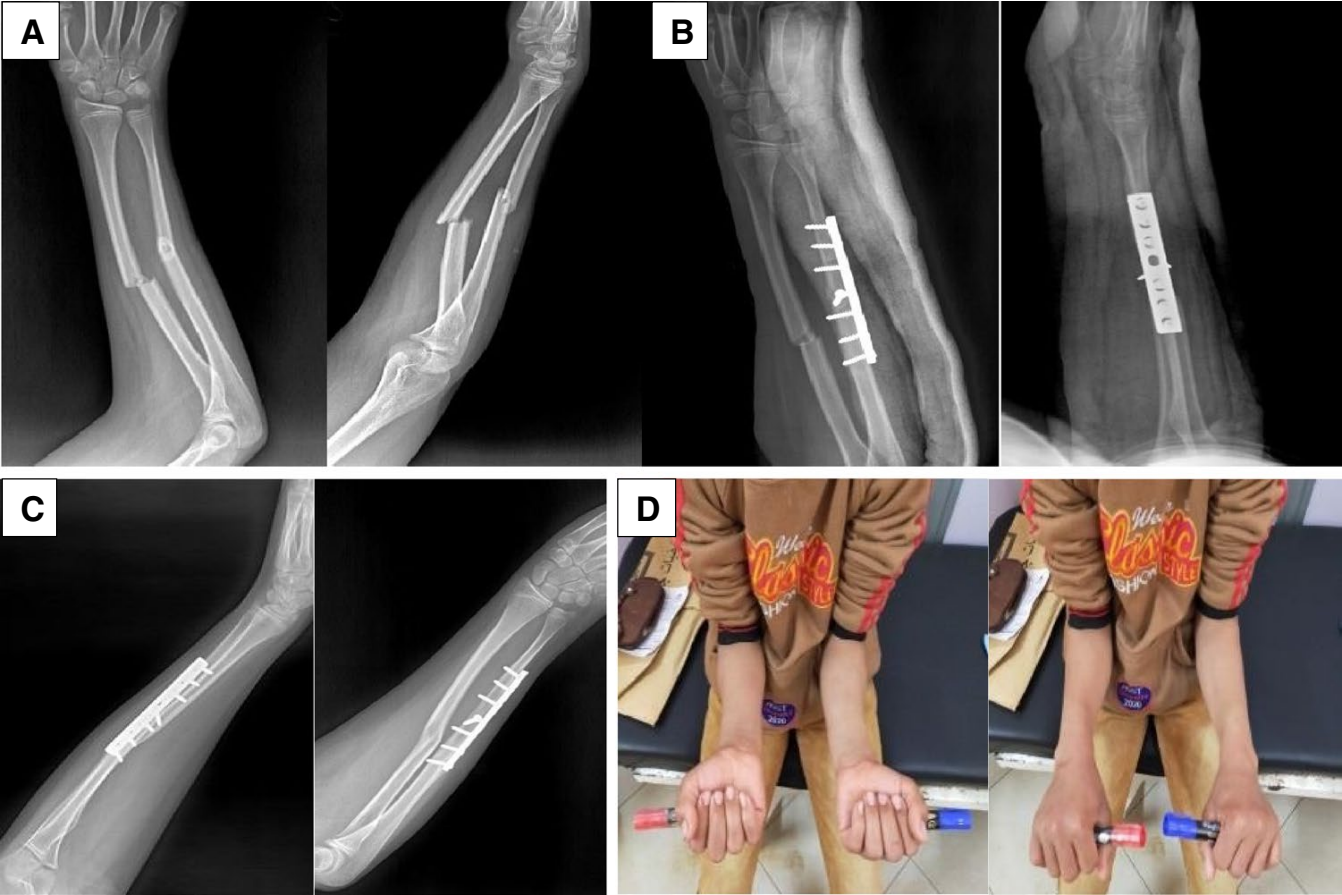
Fourteen-year-old male child diagnosed with right both-bone unstable forearm injury following falling on outstretched hands. **A**—Pre-operative X-rays (AP & lateral) showing mid shaft forearm fracture with apex volar angulation. **B**—Post-operative X-rays (AP & lateral) showing single-bone ulna fixation with well reduced radius. **C**—X-rays taken at 6 weeks post-operatively confirm radiological union. **D**—Clinical photograph showing comparable forearm range of motion

**Fig. 4:**
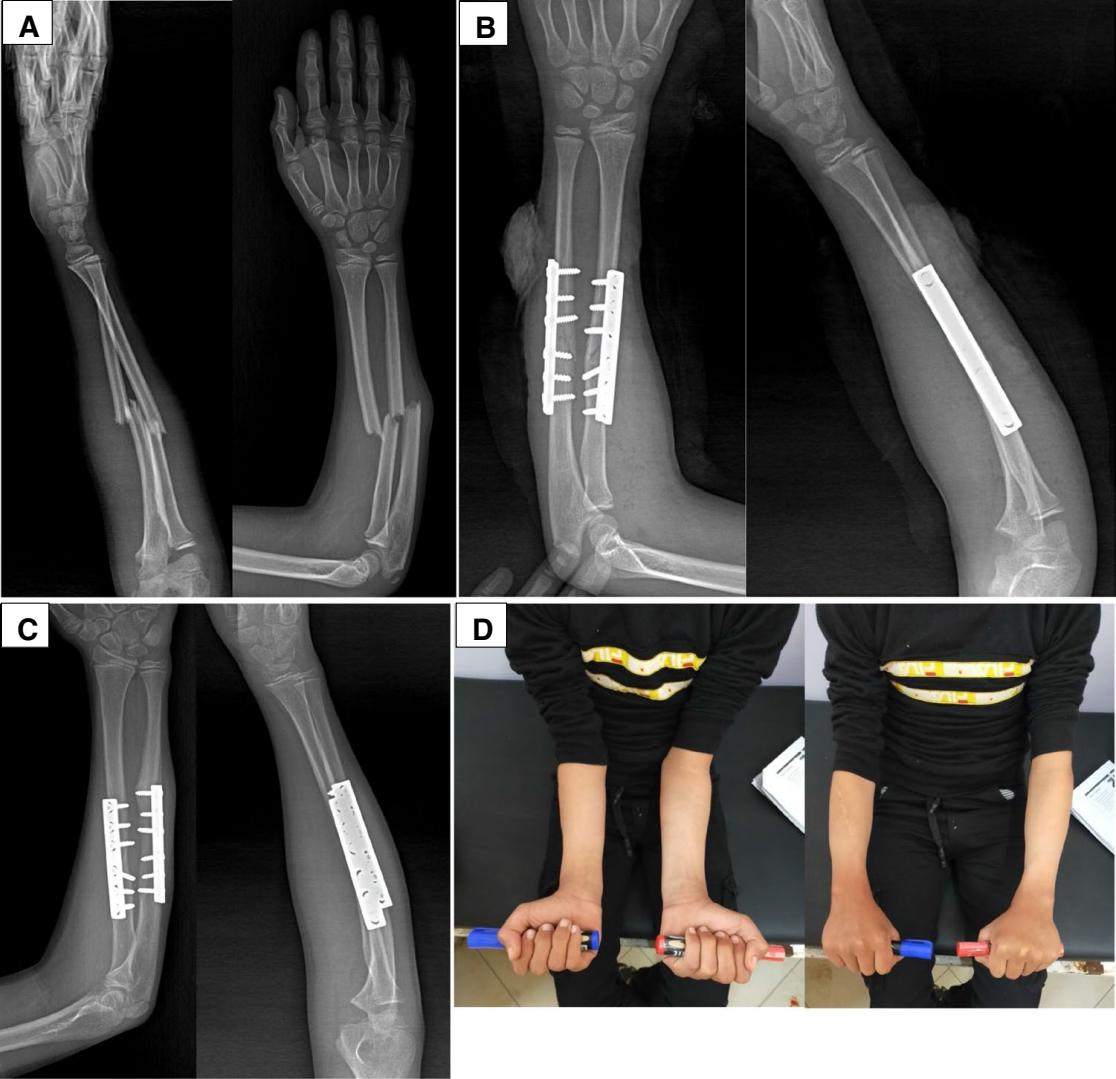
Fourteen-year-old male child diagnosed with right both-bone unstable forearm fracture following falling on outstretched hands. **A**—Pre-operative X-rays (AP & lateral) showing mid shaft forearm fracture with shortening. **B**—Post-operative X-rays (AP & lateral) showing both-bone fixation. **C**—X-rays taken at 6 weeks post-operatively confirm radiological union. **D**—Clinical photograph showing comparable forearm range of motion

The current study shows that single-bone ORIF had a higher risk of radius re-angulation than both-bone ORIF. However, the magnitude of radial re-angulation was 10° or less in 24 (96%) out of the 25 children who underwent ulna only ORIF, which falls within acceptable alignment range [5]. Only 1 (4%) patient had a radius re-angulation of 20°. This ten year-old boy had minimal limitation of less than 15° in supination/pronation ROM of the forearm. He achieved union at five week post-operatively and had an excellent price grade. Despite the higher radius re-angulation in the single-bone ORIF group, there were no clinically or statistically significant differences in ROM, union rate, time to union, or price grade in comparison to both-bone ORIF group (Figs. 2, 3, and 4). In other words, the re-angulation magnitude was not severe enough to affect the ROM, union, or function. Our findings are supported by other studies on single-bone fixation. Bhaskar and Roberts reported slight angulation of the unfixed radius following DCP plating of the ulna alone but there was no difference in functional outcome when compared to plating both bones [23]. Similarly, Hammad et al. reported favorable outcome of 18 cases of ulnar plating [24]. They reported no non-unions and all children had either a good or excellent outcome on price grading. They reported a mean loss of 12° of pronation and 5° of supination. There was a mean of 5.8° in AP angulation of the radius [24].

Although both-bone fixation can provide accurate fracture reduction, the soft-tissue exposure required can lead to complications such as infection, neurovascular injuries, scarring, and delayed union or non-union [5]. We believe that choosing the ulna for single-bone fixation further reduces soft tissue trauma due to the subcutaneous anatomy of the midshaft ulna in comparison to dorsal or volar approaches to the radius. Moreover, a single relatively smaller scar might be psychologically better for growing children and their families. Another clear advantage of single-bone fixation is shorter duration of the surgery. This translates to a shorter tourniquet time and possibly lower risk of infection. Using one implant instead of two reduces the costs of the surgery. Also, better utilization of the operation room time further reduces the costs.

While most of the studies on single-bone fixation reported encouraging results, a well designed, yet possibly underpowered, RCT by Colaris et al. reported unfavorable outcomes of single-bone fixation [9]. They reported that 67% of children had limitations to supination/pronation, and four out of 13 (30.8%) children in the single-bone group had displacement of the non-fixed bone. They also reported a high rate of non-union and refractures However, their median supination and pronation limitation at two months post-operatively (25°) significantly improved at nine months post-operatively (10°) [9]. We believe that the discrepancy between Colaris et al. findings and ours stem from the difference in the implants used; intramedullary elastic nails and DCP plates, respectively. The narrow medullary canal of forearm bones precludes inserting two nails into each bone. Even when both the radius and ulna are fixed, the curvature of both nails is in the same direction, affecting the stability [9]. Fixing one bone means that all the displacing forces are to be counteracted by one nail, making failure more likely [9]. Plating the ulna, on the other hand, achieves anatomical reduction, allows for improved fracture compression, restores the length of the forearm, and provides a stable strut for closed manipulation of the radius fracture [25]. A biomechanical study by Jones et al. suggested that ulnar plating alone can provide adequate stability when both forearm bones are fractured [26].

It is paramount to point out that ORIF of either a single-bone or both-bones are not the only options for management of unstable forearm fractures in older children. A popular and safe alternative is the use intramedullary elastic nails to fix both radius and ulna. These nails have the advantage of being inserted percutaneously. A comparative study between elastic nails and plates by Smith et al. showed that there is higher complication rate with elastic nails than with plates, 42% vs. 33%, respectively. However, most of these complications were minor [27]**.** Another retrospective study by Reinhardt et al. concluded that plates and nails have a similar capacity to restore radial bow and an equivalent union rate and forearm rotation [28]. Interestingly, seven (36.8%) out of 19 patients who underwent elastic nailing required open reduction due to inability to pass the nail in the latter study [29]. However, fixation of a single-bone using nails is not recommended based on the findings of Colaris et al. as discussed earlier.

### Limitations

This study is not without its limitations. First, there was a small number of patients in either group. The prevalence of unstable paediatric both bones fracture remains low, which is reflected by the small number of children in similar studies [9, 12–14, 16–19]. At our tertiary university medical centre, out of 345 paediatric forearm fractures that presented to the emergency department, only 64 were unstable enough to merit operative intervention. While this study might have been underpowered, the required numbers suggested by the power calculation were successfully recruited.

Moreover, the measurement of pronation/supination was done by one senior author, which could have possibly affected the accuracy of the measurement. However, measuring pronation/supination using a goniometer has been reported to have excellent inter and intraobserver reliability [29]. Additionally, grip strength was measured clinically comparing both hands due to logistics and unavailability of a more objective instrument at our centre.

Furthermore, our results might have limited applicability to other fixation implants and different postoperative protocols. We achieved an anatomical reduction and absolute stability of the fractured ulna using plates, with lag screws occasionally used. Also, the single-bone fixation group was immobilized in a backslab for four weeks post-operatively. Caution should be practiced if single-bone fixation is adopted using elastic intramedullary nails as the results can be unfavorably different, as discussed earlier [9].

Finally, the follow-up duration in our clinical was six months. Our medical centre provides care to a large geographic area in South XX, and longer follow-up duration would burden the families from rural and remote areas. Consequently, we are unable to arrive at solid conclusions on refractures and final alignment as the fractures remodel.

## Conclusion

Single-bone ulna open reduction and plate fixation and casting are safe and had a significantly shorter operative time than both-bone fixation. However, single-bone ORIF had a higher risk radius re-angulation, alas clinically acceptable. Both groups had equally excellent functional outcomes, forearm ROM, and union rates with no complications or refractures. Long-term studies are required.

## Data Availability

Not applicable.
